# In Vitro Validation of a Novel Continuous Intra-Abdominal Pressure Measurement System (TraumaGuard)

**DOI:** 10.3390/jcm12196260

**Published:** 2023-09-28

**Authors:** Salar Tayebi, Robert Wise, Ashkan Zarghami, Luca Malbrain, Ashish K. Khanna, Wojciech Dabrowski, Johan Stiens, Manu L. N. G. Malbrain

**Affiliations:** 1 Department of Electronics and Informatics, Vrije Universiteit Brussel, 1050 Brussels, Belgium; salar.tayebi@vub.be (S.T.); ashkan.zarghami@vub.be (A.Z.); jstiens@etrovub.be (J.S.); 2 Adult Intensive Care, John Radcliffe Hospital, Oxford University Hospitals Trust, Oxford OX3 7LE, UK; rob.wise@ouh.nhs.uk; 3 Discipline of Anaesthesia and Critical Care, School of Clinical Medicine, University of KwaZulu-Natal, Durban 4000, South Africa; 4 Faculty of Medicine and Pharmacy, Vrije Universiteit Brussel (VUB), 1090 Brussels, Belgium; 5 Faculty of Medicine, Katholieke Universiteit Leuven, 3000 Leuven, Belgium; luca.malbrain@hotmail.com; 6 Wake Forest University School of Medicine, Atrium Health Wake Forest Baptist Medical Center, Winston-Salem, NC 27106, USA; ashish@or.org; 7 Outcomes Research Consortium, Cleveland, OH 44106, USA; 8 Perioperative Outcomes and Informatics Collaborative (POIC), Winston-Salem, NC 27106, USA; 9 First Department of Anaesthesiology and Intensive Therapy, Medical University of Lublin, 20-954 Lublin, Poland; w.dabrowski5@yahoo.com; 10 Medical Data Management, Medaman, 2440 Geel, Belgium; 11 International Fluid Academy, 3360 Lovenjoel, Belgium

**Keywords:** intra-abdominal pressure, intra-vesical pressure, intra-gastric pressure, bladder fill volume, temperature dependency, error grid analysis

## Abstract

**Introduction:** Intra-abdominal pressure (IAP) has been recognized as an important vital sign in critically ill patients. Due to the high prevalence and incidence of intra-abdominal hypertension in surgical (trauma, burns, cardiac) and medical (sepsis, liver cirrhosis, acute kidney injury) patients, continuous IAP (CIAP) monitoring has been proposed. This research was aimed at validating a new CIAP monitoring device, the TraumaGuard from Sentinel Medical Technologies, against the gold standard (height of a water column) in an in vitro setting and performing a comparative analysis among different CIAP measurement technologies (including two intra-gastric and two intra-bladder measurement devices). A technical and clinical guideline addressing the strengths and weaknesses of each device is provided as well. **Methods:** Five different CIAP measurement devices (two intra-gastric and three intra-vesical), including the former CiMON, Spiegelberg, Serenno, TraumaGuard, and Accuryn, were validated against the gold standard water column pressure in a bench-top abdominal phantom. The impacts of body temperature and bladder fill volume (for the intra-vesical methods) were evaluated for each system. Subsequently, 48 h of continuous monitoring (*n* = 2880) on top of intermittent IAP (*n* = 300) readings were captured for each device. Using Pearson’s and Lin’s correlations, concordance, and Bland and Altman analyses, the accuracy, precision, percentage error, correlation and concordance coefficients, bias, and limits of agreement were calculated for all the different devices. We also performed error grid analysis on the CIAP measurements to provide an overview of the involved risk level due to wrong IAP measurements and calculated the area under the curve and time above a certain IAP threshold. Lastly, the robustness of each system in tracking the dynamic variations of the raw IAP signal due to respirations and heartbeats was evaluated as well. **Results:** The TraumaGuard was the only technology able to measure the IAP with an empty artificial bladder. No important temperature dependency was observed for the investigated devices except for the Spiegelberg, which displayed higher IAP values when the temperature was increased, but this could be adjusted through recalibration. All the studied devices showed excellent ability for IAP monitoring, although the intra-vesical IAP measurements seem more reliable. In general, the TraumaGuard, Accuryn, and Serenno showed better accuracy compared to intra-gastric measurement devices. On average, biases of +0.71, +0.93, +0.29, +0.25, and −0.06 mm Hg were observed for the CiMON, Spiegelberg, Serenno, TraumaGuard, and Accuryn, respectively. All of the equipment showed percentage errors smaller than 25%. Regarding the correlation and concordance coefficients, the Serenno and TraumaGuard showed the best results (R^2^ = 0.98, *p* = 0.001, concordance coefficient of 99.5%). Error grid analysis based on the Abdominal Compartment Society guidelines showed a very low associated risk level of inappropriate treatment strategies due to erroneous IAP measurements. Regarding the dynamic tracings of the raw IAP signal, all the systems can track respiratory variations and derived parameters; however, the CiMON was slightly superior compared to the other technologies. **Conclusions:** According to the research guidelines of the Abdominal Compartment Society (WSACS), this in vitro study shows that the TraumaGuard can be used interchangeably with the gold standard for measuring continuous IAP, even in an empty artificial bladder. Confirmation studies with the TraumaGuard in animals and humans are warranted to further validate these findings.

## 1. Introduction

Intra-abdominal pressure (IAP) is the steady-state pressure within the abdominal compartment, and it has been recognized as another vital sign in critically ill patients [1,2,3,4]. IAP equal to or higher than 12 mm Hg is defined as intra-abdominal hypertension (IAH) and plays an important role in the morbidity and mortality of patients admitted to the intensive care unit (ICU) [5]. According to previous studies in mixed ICU patients, slightly more than 50% of patients develop IAH within the first week of ICU admission [6,7]. Other studies have also shown high IAH prevalence following cardiac surgery (31.8%), burns (53%), trauma (36%), liver cirrhosis (82.1%), severe acute pancreatitis (72.2%), general surgery (67.3%), and sepsis (43.5%) [8,9,10,11,12,13,14]. During IAH, higher external pressure is applied to the abdominal blood vessels, which in turn results in reduced venous return, lower cardiac output (CO), and perfusion pressure, and finally, results in a vicious cycle of multiple organ dysfunction and failure depending on the magnitude and duration of IAH [15]. Therefore, early IAH detection, and subsequently, proper patient care and management to prevent abdominal compartment syndrome (ACS) has the potential to improve treatment efficiency and shorten ICU stays [16].

Clinical examination is inaccurate for estimating IAP and has a sensitivity of only 40% [4]. Other methods to measure IAP include direct IAP assessment through a peritoneal catheter (e.g., during CAPD or laparoscopy) [17] and indirect catheter-based IAP monitoring through an abdominal organ (i.e., intra-bladder, intra-gastric, intra-rectal, intra-uterine, via the femoral vein, etc.) [18,19,20,21]. Other non-invasive IAP measurement alternatives have been suggested as well. For instance, IAP estimation based on biomarkers [22], ultrasonography [23,24], tensiometry [25], bio-impedance [26], and microwave-based techniques [27,28] have shown a promising future but need to be investigated further. 

Direct IAP measurement through the peritoneal space is rather invasive and carries a high risk of infection and trauma or bleeding. Biomarkers are promising; however, they are rather unspecific and can be influenced by other pathologies as well. Other less and non-invasive methods are still in the research and development stage and have not been commercialized yet. Currently, IAP evaluation through the bladder is the gold standard, advocated for by the Abdominal Compartment Society (WSACS, formerly known as the World Society of Abdominal Compartment Syndrome, www.wsacs.org (URL accessed on 13 July 2023) and www.wsacs.mn.co (URL accessed on 13 July 2023)) [29]. This gold standard, which is also known as intra-vesical or intra-bladder pressure measurement, should be performed in the supine position at end-expiration after instilling a maximum of 25 mL of saline into the bladder, with the zero-reference level where the mid-axillary line crosses the iliac crest [29].

The aim of the present study was to validate in vitro a novel continuous IAP (CIAP) monitoring device, the TraumaGuard (Sentinel Medical Technologies, Jacksonville, FL, USA), against the gold standard height of the water column in a bench-top abdominal phantom. The new device was compared with previously validated techniques, including two intra-gastric and two intra-vesical CIAP monitoring tools: respectively, the CiMON (formerly Pulsion Medical Systems, now integrated into Getinge, Sölna, Sweden), Spiegelberg (Spiegelberg, Hamburg, Germany), Serenno (Serenno Medical, Yokne’am Illit, Israel), and Accuryn (Potrero Medical, Hayward, CA, USA). Using the bench-top phantom, we compared these technologies to each other and the preset IAP level in the phantom (via the height of the water column as the IAP reference gold standard). The outcomes of this research could serve as guidelines for using these monitoring technologies in critically ill patients. Furthermore, the overview of the strengths and weaknesses of each type of equipment could help clinicians select the most suitable technology based on the additional parameters to be monitored continuously. 

## 2. Materials and Methods

### 2.1. TraumaGuard System

The TraumaGuard (TG) intra-abdominal pressure-sensing catheter, developed by Sentinel Medical Technologies (SMT, Jacksonville, FL, USA), is an innovative urine drainage catheter that provides continuous biometric monitoring of the IAP and core body temperature (CBT). Since no previous studies on this technology have been performed, the TraumaGuard is explained in detail.

The TG is a silicone Foley catheter with scientifically coated polyurethane sensing balloons to prevent osmosis and loss of pressure over time. The TG has a urine drainage lumen, an inflation channel, and an integrated retention balloon about 5 cm from the tip of the catheter. The catheter has an atraumatic polished tip with two opposite drainage eyes in between the silicone and polyurethane balloons, avoiding contact with the bladder wall and trauma, and thus, decreasing the risk of urinary tract infections over time. There are 2 polyurethane balloons used to detect changes in the IAP: the outer distal balloon and the inner sensing balloon. The outer balloon is filled with 3 mL of sterile water to ensure contact with the bladder wall in an empty bladder. The inner sensing balloon has an air column that runs the entire length of the catheter from the middle of the outer sensing balloon to a transducer in the hub. The TG, in conjunction with the TG cable, connects to any ICU hospital bedside monitor to record the continuous IAP and CBT. The inflation, drainage, and sensing ports of the catheter are color-coded and can be operated with any 10 mL syringe tip. The zeroing and start-up procedure is explained in detail below:

- Connect the cable to the patient’s bedside monitor.
- Monitor that the atmospheric pressure equal to zero is shown.
- The LED on the cable should be white. If the LED is red, disconnect the cable from the patient’s bedside monitor and follow steps A–B. If the LED on the cable remains red, do not use the cable, and follow steps A–B with another TG cable.
- With the patient in the supine position, inflate the outer distal balloon:
  - Connect a syringe with 6 mL of sterile water to the outer distal balloon valve (white port).
  - Pull the vacuum to evacuate excess air from the manufacturing process.
  - Inflate the outer distal balloon with exactly 6 mL of sterile water.
- With the patient in the supine position, connect the TG cable to the TG catheter.
  - Insert the TG cable into the TG catheter by lining up two pins on the TG cable side, with two openings on the catheter side.
  - Insert until firmly in place.
  - Twist the locking mechanism clockwise until secure.
  - The LED on the cable should be white.
- Reduce the volume in the outer distal balloon.
  - Remove exactly 3 mL of sterile water from the outer distal balloon via the syringe on the outer distal balloon valve (white port).
  - Disconnect the syringe.
- The monitor should now display the real-time unfiltered IAP with the patient in the supine position.
- To enter the supine filter mode, which gives the end-expiratory pressure, press the capture button on the cable for 3 s. The LED on the cable should be green.

### 2.2. CiMON System

The CiMON technology (formerly Pulsion Medical Systems, now integrated into Getinge, Sölna, Sweden) is equipped with a regular nasogastric feeding probe (5.3 mm outer diameter), which is connected to an inflatable balloon, which can be filled with a maximum of 1.1 mL air and has been extensively described in the past [30]. The balloon is then connected to an external monitoring system (the CiMON monitor) with autocalibration (once every hour), and pressure measurement algorithms. The measurement process is based on air compression inside the air-filled balloon. Therefore, the IAP can be determined continuously and automatically according to the pressure transmitted to the monitoring device from the air compression inside the balloon. The autocalibration mode was turned off during the experiment.

### 2.3. Spiegelberg System

The Spiegelberg (Spiegelberg, Hamburg, Germany) is an air-capsule-based measurement device, which has been described previously [31]. This technology, similar to the CiMON, consists of a nasogastric tube-like catheter with a 3 mm outer diameter that is connected to an air-filled balloon with a total filling volume of 0.1 mL. The IAP measurement is also similar to the CiMON and equal to the pressure needed to compress the air inside the balloon. The autocalibration process is performed once every hour. The autocalibration mode was turned off during the experiment.

### 2.4. Serenno System

The Serenno device is a novel technology to measure IAP in addition to urine output, and it has been studied in previous studies in vitro and in patients undergoing laparoscopy [18]. This system consists of a control unit and a disposable component. The disposable unit is a special pressure-sensing fluid pump that is connected in a series between the Foley catheter and the urine collection bag. The disposable unit is also connected via air tubes to a controller unit that measures the IAP automatically. The controller operates and collects data from the disposable unit by measuring and controlling the air pressure in each of the air tubes in a sequential procedure to measure both the IAP and urine output. 

### 2.5. Accuryn System

Potrero Medical (Hayward, CA, USA) has developed and patented an innovative technology to measure the pressure in body compartments, such as the bladder [32]. This platform technology can be used in multiple segments within (critical care) medicine. The Accuryn SmartFoley and Monitoring System (Potrero Medical, Hayward, CA, USA) eliminates standing urine in the bladder and drainage system via an active drain line clearance and three one-way valves to eliminate urinary backflow. These features help to prevent retained urine (due to airlocks), reduce false oliguria, and enable accurate CIAP and continuous urinary output. The Accuryn System is an entirely closed system wherein the actual urinary catheter is connected to the tubing system, which is further secured by plastic wrapping. This closed system is interlocked into the monitoring system without any further intervention necessary on the drainage system itself. Bladder pressure, as a surrogate of IAP, is measured through a semi-flaccid balloon containing a pressure sensor at the tip of the urinary catheter. Potrero Medical has focused development efforts on the Accuryn Catheter System for IAP measurements, which are required to detect IAH and ACS. 

### 2.6. Abdominal Phantom

**Figure 1:**
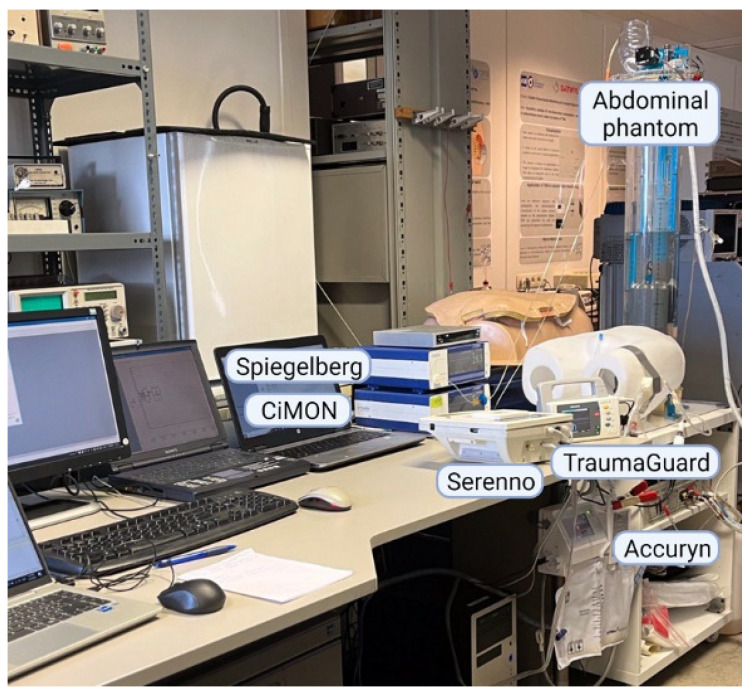
Study setup. CiMON and Spiegelberg nasogastric catheters were placed at the bottom of the water tank. Dedicated TraumaGuard and Accuryn Foley catheters were inserted inside the artificial bladder at the bottom of the abdominal phantom. The Sentinel Medical smart cable was then connected to the TraumaGuard catheter.

Since the abdominal compartment is fluid-based, it follows the hydrostatic laws of Pascal [33]. Therefore, IAP can be simulated using a water chamber (containing an artificial bladder), with the possibility of changing the water height on top of the water chamber to adjust the superimposed IAP. Taking this concept into account, a human phantom was designed and used in this study to validate and compare different technologies to the reference standard, which was the water height of the water column in the abdominal phantom. The phantom has been extensively described previously and consists of a controller unit (via a laptop), which provides the possibility of adjusting the IAP by changing the height of the water column [34]. At the bottom of the water chamber, there is a 500 mL balloon (Ambubag, Grevenberg–Dahlhausen, Bochum, Germany), which acts as an artificial bladder and contains Foley catheters. For the purpose of this study, the TraumaGuard and Accuryn Foley catheters were inserted next to each other, and the Serenno disposable device was then connected to the TraumaGuard catheter, as the Accuryn is a closed system. The artificial bladder can be filled with continuous fluid irrigation and drainage to simulate urine production. In addition to the IAP, by using two extra moving cylinders of different sizes (at different frequencies) in the water chamber, respiration rate and heartbeats can be simulated as well. Figure 1 shows the final setup used in the present study. 

### 2.7. Study Design

The CiMON and Spiegelberg systems were connected to a balloon-tipped catheter. The balloons at the tip of the catheters were placed at the zero level of the water column. The Serenno, Accuryn, and TraumaGuard systems were connected to a dedicated Foley catheter, and subsequently, to the artificial bladder at the bottom of the water column. Several scenarios were designed to examine the robustness of each device to measure and monitor the IAP continuously. 

The impacts of the bladder fill volume and the fluid (body) temperature were evaluated first. The water height was kept constant at 15 mm Hg in the abdominal phantom. The IAP was then measured with different fluid volumes inside the artificial bladder. The fluid volume inside the bladder was increased from 0 to 20 mL in steps of 1 mL, from 20 to 200 mL in steps of 10 mL, from 200 to 400 mL in steps of 50 mL, and lastly, from 400 to 500 mL in steps of 10 mL. Using this approach, the most optimal fill volume of the bladder was determined. The known maximal volume of the artificial bladder (as per the company’s description) was 500 mL. During the next step, the temperature dependency was examined for each device; the height of the water column inside the abdominal phantom was increased from 5 to 35 mm Hg (with stepwise increases of 5 mm Hg), and the IAP measurements were performed at room (20 °C) and body temperature (37 °C) and compared. 

After the assessment of the temperature and bladder fill volume impact on each technology, we investigated the correlation coefficient, accuracy, bias, precision, limits of agreement, percentage error, and concordance of each device by simulating different IAP values between 0 and 40 mm Hg. In order to have a good analysis of each device, we analyzed not only the intermittent IAP readings (*n* = 300) but also the continuous IAP for a period of 48 h, with an IAP average taken every minute (*n* = 2880). The intermittent IAP and CIAP readings obtained with each system were compared with the reference values of the abdominal phantom. For the first time, we also performed error grid analysis for each system to determine the risk levels involved with wrong treatment strategies due to erroneous CIAP measurement. This analysis has been used in the past for other measurement technologies, including blood glucose and arterial pressure measurements [35,36]; however, no similar analysis has been carried out previously for CIAP measurement devices. For each 12 h recording period, we calculated the area under the curve (AUC) and the time above a certain threshold (TAT, <12; 12–15; 16–20; 21–25; >25 mm Hg). 

Lastly, the ability of each measurement device to detect dynamic IAP variations due to respiration and heartbeat artifacts was assessed by simulating respiration and heartbeats through the abdominal phantom. Applying a respiration rate of 15 rpm and a heartbeat rate of 120 bpm, the dynamic tracings of each system were obtained. Consequently, the end-inspiration IAP (IAP_ei_, or the highest value), end-expiration IAP (IAP_ee_, or the lowest value), ΔIAP (defined as IAP_ei_ minus IAP_ee_), mean IAP (defined as the average of IAP_ei_ and IAP_ee_), abdominal pressure variation (APV, defined as ΔIAP divided by the mean IAP), heart rate (HR), respiration rate (RR), and inspiration-to-expiration ratio (I/E) were extracted from the dynamic raw IAP data. 

### 2.8. Raw Data Processing

Any possible offset due to the height difference between the zero level of the water column and the position of the tip or balloon of the bladder catheter in the artificial bladder, and the position of the measurement balloon at the tip of the nasogastric catheters were adjusted. Re-sampling and interpolation of the recorded data was the second step of signal pre-processing of the time series data since every technology had its own sampling rate to capture the raw IAP data. Therefore, all the recorded traces were resampled to provide the IAP readings per minute. Lastly, for the height of the water column inside the abdominal phantom, the continuous tracings were generated based on the 300 intermittent readings and using linear interpolation to convert the discrete IAP readings to a continuous trend of 48 h. Afterward, a first-order median filter was used to remove any non-ideality (spark noise) of each signal and to improve the tracings before further analyses. After each filtering, the processed data were compared with the raw data to make sure that the original recorded values had not been changed. Signal alignment was the next step—the timings of all the tracings were adjusted with respect to each other to make sure that they represented the IAP values at the same time intervals. 

### 2.9. Statistical Analysis

After signal pre-processing, filtering, and alignment, Pearson’s correlation analysis was used to calculate the correlation coefficient (R) between each pair of the measurements (the new device or IAP_device_ vs. the gold standard height of the water column or IAP_H2O_). Additionally, Lin’s concordance correlation analysis was used to calculate the concordance correlation coefficient (CCC) and to assess the alignment between the signals. In fact, Pearson’s correlation analysis evaluated the robustness of the linear association between signals (precision), while Lin’s correlation analysis examined the agreement of two signals, taking the 45° line through the origin into account (precision and accuracy) [37]. Two methods were considered equal if the line of identity crosses the origin of the X- and Y-axis, and if the R^2^ was greater than 0.6. The consistency among the measurement technologies was evaluated by means of intra-class correlation (ICC) analysis. An ICC coefficient smaller than 0.5 shows poor consistency among the recorded data, while values between 0.5 and 0.75, 0.75 and 0.9, and above 0.9 show moderate, good, and excellent consistency and reliability, respectively [38]. The bias of each system was defined and calculated as the mean difference between the IAP_H2O_ and the IAP_device_. Subsequently, the precision and limits of agreement were defined as the standard deviation of the bias and the bias ±1.96 times the precision according to Bland and Altman [39]. The percentage error was then calculated as the precision multiplied by two and divided by the mean IAP value of each technology. The ability of each technology to track changes in the IAP (ΔIAP) over time was evaluated by means of concordance analysis. In fact, the ΔIAP of each device was measured and plotted versus the ΔIAP_H2O_ in the same time interval. The concordance coefficient was then defined as the percentage of pairs with the same direction of change after the exclusion of pairs with both a ΔIAP_device_ and ΔIAP_H2O_ ≤ 2.5 mm Hg (or less than 15% of change) and the exclusion of pairs with either the ΔIAP_device_ or ΔIAP_H2O_ equal to zero [40]. Error grid analysis was also carried out for each device to assess the risk level for a wrong treatment strategy due to erroneous IAP measurements by each device, as described previously for continuous blood glucose and arterial pressure monitoring [35,36]. Taking the guidelines of the Abdominal Compartment Society into account, different risk regions were defined according to the IAH grade [29]. The examined IAP range of 0 to 40 mm Hg was divided into sub-regions of 0–11 mm Hg, 12–15 mm Hg, 16–20 mm Hg, 21–25 mm Hg, and 26–40 mm Hg. The no-risk region was then defined as the areas where both the IAP_device_ and IAP_H2O_ showed the same IAH grade. The low-risk regions, on the other hand, were defined as the areas where the IAP_device_ and IAP_H2O_ showed two consecutive IAH grades. In a similar way, the medium- and high-risk regions were defined as the areas where the IAP_device_ and IAP_H2O_ showed two and three IAH grade differences between each other, respectively. Statistical analysis was performed with Excel (Microsoft Excel 2007, Microsoft Corporation, Redmond, WA, USA), Statistical Package for the Social Sciences (SPSS Inc., Chicago, IL, USA), and MATLAB (The MathWorks Inc., Natick, MA, USA).

## 3. Results

### 3.1. Impact of Bladder Fill Volume 

**Figure 2:**
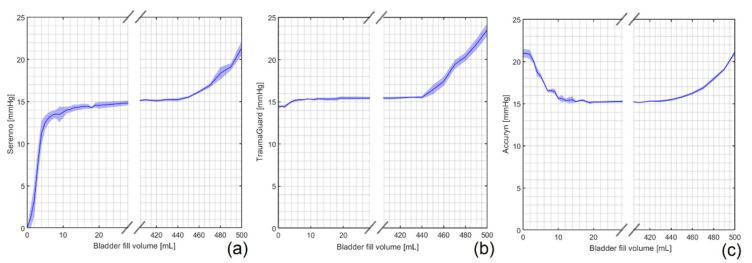
Impact of bladder fill volume on intra-abdominal pressure during 5 repeated measurements at a preset gold standard IAP of 15 mm Hg. IAP results obtained with (**a**) Serenno, (**b**) TraumaGuard, and (**c**) Accuryn. As can be seen, the Serenno and Accuryn, respectively, under- and overestimated the IAP values when the bladder fill volume was less than 15 mL, approximately. All of the systems exhibited significantly higher IAP values when the bladder was fully filled (fluid volume of more than 440 mL). The area between 30 and 400 mL has been removed since the IAP value was constant in this interval.

The artificial bladder of the phantom in the empty and fully filled conditions is shown in ESM Figure S1. As illustrated in Figure 2, the Serenno (underestimation) and Accuryn (overestimation) systems showed erroneous IAP values when the bladder fill volume was between 0 and 15 mL, approximately. The TraumaGuard device was, however, able to measure the IAP even in an empty bladder. 

The artificial bladder used in this study had a total volume of 500 mL. As can be observed in Figure 2, when the fluid volume inside the bladder was higher than 440 mL, the measured IAP values were not representative anymore for the surrounding gold standard pressure. For the purpose of the study, the bladder fill volume was maintained at around 200 mL throughout the entire experiment.

### 3.2. Fluid (Body) Temperature

The next studied parameter was the impact of the fluid (body) temperature on each system. The temperature of the abdominal phantom was measured by the temperature sensor of the Foley catheter. The Spiegelberg was the only equipment that showed a change in the IAP values when the temperature was increased abruptly. Although the mean difference between the measured IAP by the gold standard and the Spiegelberg for pressures from 5 to 35 mm Hg was only 1.85 ± 0.56 mm Hg at 20 °C, it increased significantly to 5.78 ± 4.88 mm Hg at 37 °C. However, after simple recalibration of the device, it again performed the IAP measurements in the correct way, also at 37 °C.

From the other side, the mean bias between the water column height and the CiMON, TraumaGuard, Accuryn, and Serenno changed by 0.08 ± 0.05, 0.75 ± 0.48, 1.55 ± 0.15, and 0.1 ± 0.08 mm Hg, respectively, when the temperature was increased from 20 to 37 °C. However, the variation does not seem to be solely attributable to the increase in temperature. ESM Figure S2 shows the relative error of the IAP measurements at 20 °C and 37 °C.

### 3.3. Comparison of IAP_device_ and IAP_H2O_

**Figure 3:**
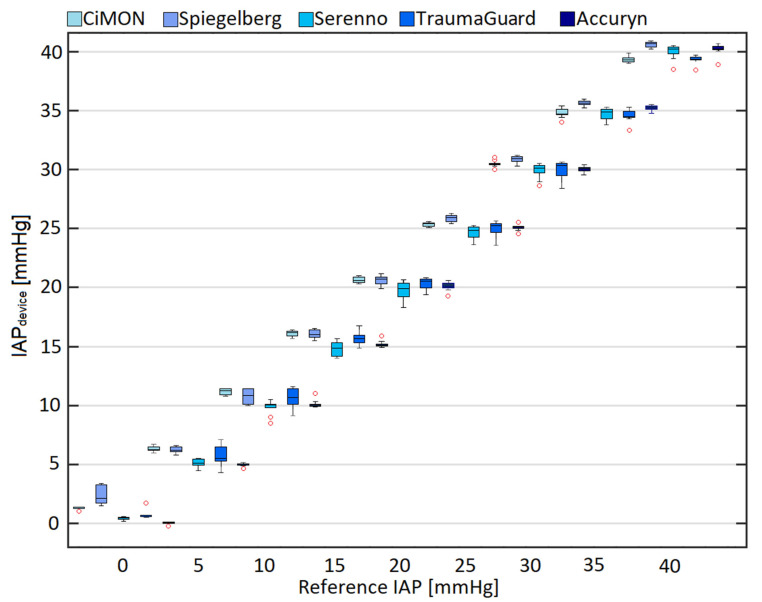
Box and whisker plots comparing IAP_H2O_ and recorded IAP_device_. The number of measurements at each IAP preset value between 0 and 40 mm Hg was 10. The error bars are the 95% confidence interval, the bottom and top of the box are the 25th and 75th percentiles, the line inside the box is the 50th percentile (median), and any outliers are shown as red circles. Analysis of variance (ANOVA) revealed a *p*-value < 0.005 among the measurement devices at preset values of 0, 10, 15, 20, 25, 30, 35, and 40 mm Hg. The mean and standard deviation of each technology at each IAP value are also presented in ESM Table S1.

After the evaluation of the potential impacts of two fundamental parameters on each technology, we calculated the mean and standard deviation of each system at different gold standard reference pressures (height of the water column). Figure 3 illustrates the comparison of the intra-abdominal pressure (IAP_device_) measurements obtained by each device at different levels of the gold standard reference (IAP_H2O_), using boxplots of 10 IAP values obtained with each device over the examined pressure range between 0 and 40 mm Hg. It can be seen that most of the systems showed a slight overestimation of the IAP at reference pressure values less than 20 mm Hg. For reference pressure values higher than 20 mm Hg, however, they either provided an underestimation or a smaller overestimation of the real IAP value.

### 3.4. Pearson’s, Lin’s, and Intra-Class Correlation Analyses

Pearson’s and Lin’s correlation analyses determined the correlation and concordance correlation coefficient between each pair of measurements. Based on the obtained results, the best correlation was observed between the IAP_TraumaGuard_-IAP_H2O_ (R = 0.99, CCC = 0.99, *p*-value = 0.001) and the IAP_Serenno_-IAP_H2O_ (R = 0.99, CCC = 0.99, *p*-value = 0.001). The Accuryn (R = 0.98, CCC = 0.97, *p*-value = 0.001), CiMON (R = 0.98, CCC = 0.97, *p*-value = 0.001), and Spiegelberg (R = 0.98, CCC = 0.96, *p*-value = 0.001) also showed a good correlation with the height of the water column as the reference gold standard. Regarding the intermittent recorded values, all of the systems showed a very good correlation with the reference gold standard (R = 0.99, *p*-value = 0.001). ESM Figure S3 shows the correlation matrix in addition to the correlation coefficients and the *p*-values for each paired measurement. 

The ICC coefficient comparing each technology with the reference method (for continuous 48 h monitoring) is presented in ESM Figure S4. In general, an excellent consistency was observed between each technology and the gold standard. However, the Serenno and TraumaGuard showed slightly higher consistency and reliability (ICC = 98.7% compared to ICCs of 98.6%, 97.7%, and 97.2% for the Accuryn, CiMON, and Spiegelberg, respectively). 

### 3.5. Bland and Altman Analysis

**Figure 4:**
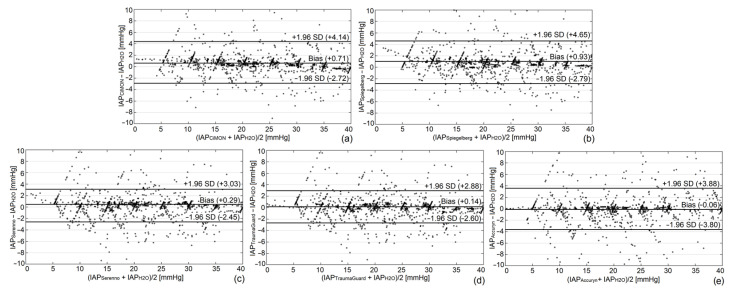
Bland and Altman plots according to 48 h of continuous tracings. Bland and Altman analysis was carried out for each system: (**a**) CiMON, (**b**) Spiegelberg, (**c**) Serenno, (**d**) TraumaGuard, and (**e**) Accuryn. The bias, precision, limits of agreement, and percentage error were calculated as well.

In order to calculate the bias, precision, limits of agreement, and percentage error, Bland and Altman analysis was used as previously described [39]. Bland and Altman graphs for the continuous IAP measurements are provided in Figure 4.

All devices performed adequately well in this in vitro setting, in line with the WSACS recommendations, and had a low bias with small limits of agreement and percentage errors, demonstrating that they can all be used interchangeably with the gold standard for both intermittent and continuous IAP monitoring. The Bland and Altman graphs for the intermittent readings are shown in the electronic supplemental material (ESM Figure S5). Table 1 shows the detailed results of the Bland and Altman analysis for each system.

### 3.6. Concordance Analysis

The next analysis performed for each device was to determine their ability to track IAP changes over time rather than solely determining the correct IAP values at one given point in time per the concordance analysis, as explained above. All devices demonstrated an excellent ability to track IAP changes. The TraumaGuard and Serenno showed the highest concordance coefficient of 99.5%, followed by the Accuryn (99.3%), CiMON (99.1%), and Spiegelberg (99.0%). The concordance graphs of each technology are shown in ESM Figures S6 and S7 for the continuous and intermittent measurements, respectively. 

### 3.7. Error Grid Analysis

**Figure 5:**
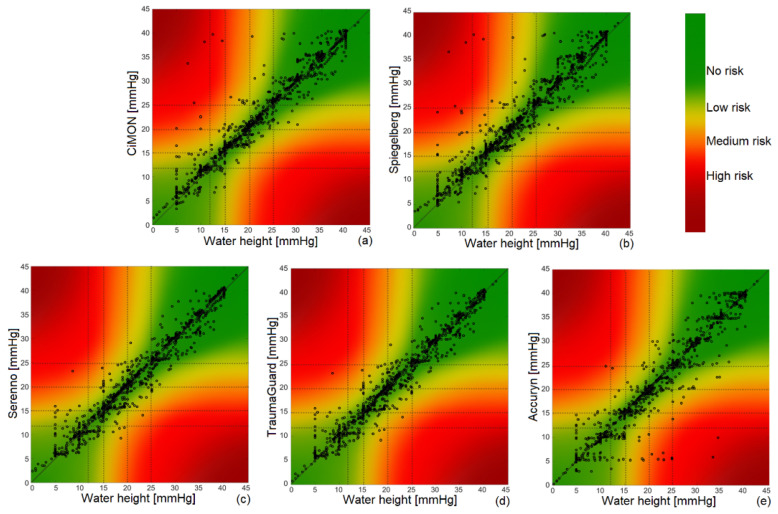
Error grid analysis for (**a**) CiMON, (**b**) Spiegelberg, (**c**) Serenno, (**d**) TraumaGuard, and (**e**) Accuryn. As can be seen, more than 92% of the measurements of each device are located in the no-risk region. Around 5%, 1%, and less than 1% of the measurements are in the low-, medium-, and high-risk regions, respectively.

Error grid analysis was carried out for each IAP measurement device to check the associated risk for wrong treatment strategies due to wrong IAP measurements. The results are summarized in Figure 5 and Table 2. 

We observed that all of the IAP monitoring devices showed very good error grid analysis results. In reviewing the results of Table 2, we can see that the Serenno and TraumaGuard showed the least risk level associated with erroneous measurements, followed by the CiMON and Spiegelberg. 

### 3.8. Analysis of Continuous Trends

**Figure 6:**
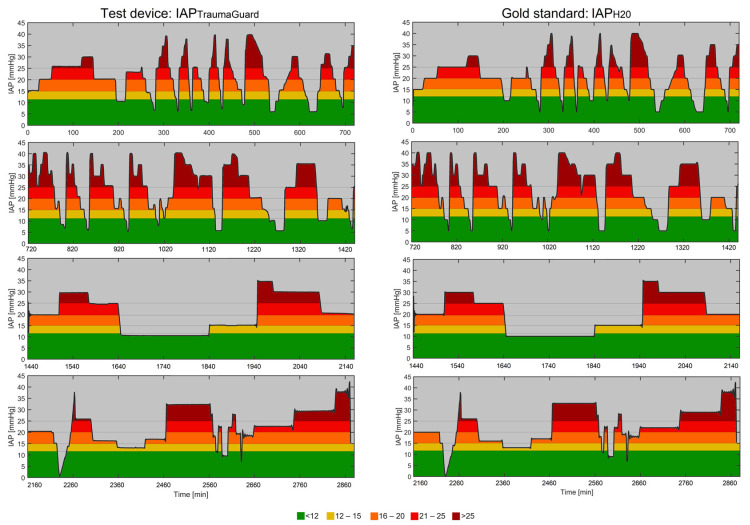
Four sets of 12 h continuous IAP measurements. The left column represents the four 12 h tracings of the TraumaGuard, while the right column represents the same time interval for the water height as the reference gold standard IAP.

Evaluation of the AUC (min × mm Hg) and TAT (min) is of great importance, as the severity and duration of the IAH plays an important role during patient monitoring in the ICU in analyzing the pressure–time burden. With dedicated software, the AUC and TAT for different IAP thresholds were calculated for the four 12 h recording periods (see Figure 6 for the TraumaGuard). The other TAT and AUC figures of the CiMON, Spiegelberg, Serenno, and Accuryn are presented in ESM Figures S8–S11.

Further numerical values regarding the TAT and AUC are also presented in Table 3.

### 3.9. Dynamic Raw IAP Tracing (Impact of Respirations and Heartbeats)

**Figure 7:**
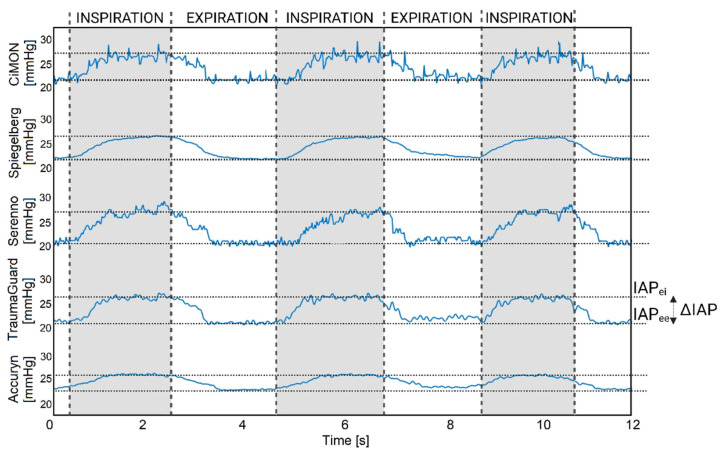
Three consecutive respiration oscillations in the IAP tracings of the CiMON, Spiegelberg, Serenno, TraumaGuard, and Accuryn. The Spiegelberg did not show enough resolution to capture the heartbeat fluctuations. Among the different devices, CiMON had the best ability in heart rate tracking. Inspiration and expiration time, IAP_ei_, and IAP_ee_ are illustrated in this figure as well.

Another important challenge for each CIAP measurement device was to track the dynamic IAP variations due to respirations and heartbeats. For this analysis, the respirations and heartbeats were adjusted and set to 15 rpm and 120 bpm, respectively. As can be observed in ESM Figure S12, all studied devices were able to track the respiration rate. Subsequently, three consecutive respiration cycles were selected for each device to have a closer look at these data, to extract further information (IAP_ei_, or the highest value; IAP_ee_, or the lowest value; ΔIAP, defined as IAP_ei_ minus IAP_ee_; mean IAP, defined as the average of the IAP_ei_ and IAP_ee_; APV, defined as the ΔIAP divided by the mean IAP; RR, and the I/E ratio), and to check the ability of the equipment to also show heartbeat variations. Figure 7 illustrates 12 s of the dynamic raw IAP variations of each device. Further derived numerical values extracted from five raw CIAP tracings are presented in Table 4.

Regarding the extracted information from the dynamic tracings of each device, it can be observed that the TraumaGuard and CiMON provided the best results for the IAP_ei_, IAP_ee_, APV, and mean IAP compared to the preset reference values. From another perspective, the Serenno, TraumaGuard, and CiMON provided the best estimation of the ΔIAP compared to the reference value of the abdominal phantom. All of the devices were able to track respiratory variations; however, heartbeat differentiation seemed more difficult due to artifacts. Although small oscillations can be seen on top of the respiration oscillations, it is difficult to understand whether this was due to the heartbeat or discrete oscillations in the water chamber of the abdominal phantom. Except for the TraumaGuard and CiMON, which showed a higher (probably related to a lag phase when the IAP decreased with expiration) and lower I/E ratio, all the other equipment determined the exact value of the inspiration-to-expiration ratio.

## 4. Discussion

To summarize our research, five devices for measuring IAP were examined. The impacts of the temperature and bladder fill volume were initially analyzed for each device. Subsequently, each device was compared to the gold standard height of the water column in a benchtop abdominal phantom, both intermittently followed by 48 h of continuous monitoring. Our results indicate that the Serenno and Accuryn systems were not able to provide correct IAP estimation when the artificial bladder was completely empty or contained less than approximately 15 mL of fluids. The Serenno showed a significant underestimation of the reference IAP value when the artificial bladder was empty. This was probably due to the study set-up, with two Foley catheters next to each other (each with a retention and sensing balloon) and the non-compliant material of the artificial bladder. The Serenno device needs fluid inside the bladder to create a water column and to transmit the intra-bladder pressure to the disposable unit, and thus, to the measurement unit. In our artificial model, we were able to drain the bladder completely and rapidly by instilling or removing volume. In real life, the Serenno will also check the urine output and allow pressure to build (resulting in a bladder volume greater than zero), allowing for correct IAP transmission. The Accuryn, on the other hand, showed a systematic overestimation of the IAP with an empty artificial bladder, which again may be related to the specific study set-up, as explained above, and does not necessarily reflect the real-life situation in a patient with a compliant bladder and the fact that even with a Foley catheter in place, the bladder may not be fully empty. Previous studies comparing a single air-charged balloon sensor with a water-filled system (gold standard) showed that the former was less accurate when the bladder was filled with less than 50 mL [41]. The TraumaGuard’s unique balloon-in-balloon technology was able to measure the IAP and track IAP changes even with an empty artificial bladder. Regarding the impact of body temperature, the Spiegelberg was the only equipment that showed an important temperature effect on the obtained IAP readings. By increasing the temperature from 20 to 37 °C, the Spiegelberg showed a significant overestimation of the reference IAP. However, after re-zeroing and calibration, the device was able to measure the IAP correctly, also at 37 °C. 

Overall, the devices analyzed in the present study exhibit a tendency to slightly overestimate a true IAP when the values were below 20 mm Hg. As the IAP reference values increased, some of the devices began to display a smaller overestimation or even an underestimation. This slight deviation can be rectified by recalibrating the technologies in situations where there is a significant change in the IAP over a short period of time. Reviewing the 48 h of continuous tracings, the TraumaGuard (bias = +0.14 mm Hg, precision = 1.4 mm Hg) and the Serenno (bias = + 0.29 mm Hg, precision = 1.4 mm Hg) showed the best interchangeability with the gold standard (lowest bias and smallest LA). However, it should be pointed out that a continuous tracing of the gold standard was generated by means of interpolation from 300 intermittent readings. Although all the technologies showed a very high correlation and concordance coefficient in comparison to the gold standard of the abdominal phantom, the Serenno (R^2^ = 0.98, *p* = 0.001) and TraumaGuard (R^2^ = 0.98, *p* = 0.001) revealed the best correlation coefficients. The error grid analysis of the IAP measurement systems also revealed the lowest risk level for the TraumaGuard, Accuryn, and Serenno. Approximately, 93% of the 2880 continuous IAP readings obtained via a Foley catheter over a period of 48 h were in the no-risk region. To the best of our knowledge, this is the first time that error grid analysis has been performed on CIAP measurement devices. The risk regions were defined based on the guidelines of the abdominal compartment society and the IAH grading [29]. In our last CIAP evaluation analysis, the studied systems were challenged to extract dynamic raw IAP variations caused by respirations and heartbeats. All devices were able to successfully track the RR, IAP_ee_, IAP_ei_, mean IAP, ΔIAP, APV, inspiratory and expiratory time, and I/E ratio; however, the CiMON showed the best overall results, especially with respect to the HR. Table 5 shows an overview of the investigated technologies. 

In general, all of the studied devices showed an excellent capability for CIAP monitoring. However, regarding the intra-gastric measurement devices, the CiMON seemed to be slightly superior compared to the Spiegelberg. The main advantages of the CiMON are the heartbeat monitoring and the low-temperature independency compared to the Spiegelberg. Both systems slightly overestimated the true IAP at all reference IAP values. Alternatively, reviewing the intra-bladder measurement devices, the TraumaGuard and Serenno appeared to be the best options, with regard to their ability to track respirations and their potential for future enhancements to measure heartbeat variations. The TraumaGuard allows for CIAP monitoring even with an empty artificial bladder due to its unique three-balloon concept. 

Overall, taking into account the research guidelines and recommendations of the Abdominal Compartment Society [42], a novel IAP measurement technology can be successfully validated against the gold standard if the bias, precision, and limits of agreement of the measurements are less than 1, 2, and 4 mm Hg, respectively. The TraumaGuard, Accuryn, and Serenno are fully interchangeable with the gold standard; however, it should be pointed out that this statement is based on the obtained results of the present in vitro study. Confirmation studies with the TraumaGuard in animals and humans are warranted to further validate these findings in different patient populations. The Spiegelberg showed a bias of 1.15 mm Hg during the intermittent readings. The gastric measurement devices also showed limits of agreement that were slightly larger than 4 mm Hg (4.14 and 4.65 mm Hg for the CiMON and Spiegelberg, respectively). Our results obtained for intermittent IAP monitoring with the CiMON and Serenno are in agreement with previously performed studies [34]. For the Spiegelberg, however, a smaller bias has been reported in previous investigations. The higher observed bias for the Spiegelberg in the present study might have been due to the altered properties of the balloon at the tip of the nasogastric catheter and not due to the measurement unit and the fact that the auto-calibration function was turned “off” during the experiment. Nevertheless, the Spiegelberg shows reliable and acceptable results sufficient for use in the clinical monitoring of ICU patients. 

Several other approaches to obtain CIAP measurements have been investigated in previous studies, such as a wireless motility capsule, direct CIAP measurement via a peritoneal catheter [43], trans-femoral venous IAP measurement [19], etc. However, the lack of sufficient accuracy, risk of infection and trauma, and poor correlation with the gold standard IAP measurements have limited the application of the abovementioned techniques. 

To conclude, measuring the IAP is a versatile tool, with clinical applications spanning multiple medical specialties, and it should be considered as the sixth vital sign next to the classic parameters, like heart rate, respiratory rate, temperature, blood pressure, and peripheral oxygen saturation. Monitoring this vital sign in critically ill patients is essential for prevention, early diagnosis, and the management of IAH and ACS [44]. Elevated IAP can impede blood flow back to the heart, leading to reduced cardiac output. This, together with increased intrathoracic pressure, may lead to increased intracranial pressure (ICP). Which, in turn, has a negative impact on cerebral perfusion pressure (CPP = MAP − ICP), potentially causing cognitive impairment, confusion, and even neurological deficits. Additionally, in patients on mechanical ventilation, elevated IAP can lead to various complications, such as decreased lung compliance, increased intrathoracic pressure, and low functional residual capacity, resulting in poor oxygenation and difficult ventilation and weaning [45]. Therefore, IAP monitoring is vital to optimize lung protective mechanical ventilation strategies and prevent ventilator-associated lung injury. Another potential application of IAP monitoring is proper hemodynamic assessment for guiding fluid resuscitation [46]. By tracking abdominal perfusion pressure (APP = MAP − IAP), healthcare providers can make informed decisions regarding hemodynamics and fluid management to maintain adequate tissue perfusion while avoiding fluid accumulation. The kidneys are highly sensitive to changes in perfusion pressure and sometimes considered canaries in the coal mine for IAH. IAH can decrease renal arterial and venous blood flow, impairing kidney function and potentially leading to acute kidney injury (AKI) [47]. This can further exacerbate the body’s fluid, acid–base, and electrolyte balance. Furthermore, since elevated IAP can indicate the presence of bleeding or organ damage, it is an important tool in the assessment and management of trauma patients [48]. 

### Limitations and Future Perspective

The research experiments were conducted using a bench-top phantom designed to simulate the human abdominal compartment. However, despite efforts to mimic the real-life situation, there may be some variations between the phantom and the actual human body. For instance, distilled water was used as a substitute for urine, which may have affected the accuracy of the findings. An artificial bladder was used that may not correspond to the real-life situation. Future research should take into account the potential influence of mass effects in the pelvic region, patient movement artifacts, posture and body position, and other factors that were not considered in this study. Additionally, the continuous tracings for water height and the Accuryn (partially) were generated by linear interpolation of the 300 intermittent readings. Therefore, in the future, it is recommended to use a data logger in order to obtain the continuous tracings automatically for the gold standard as well as the tested devices. We suggest that the methodology presented herein, including the use of error grids and the calculation of the AUC and TAT, in combination with the analysis of the derived parameters obtained from the raw IAP data, should be used as a standard for future clinical validation of CIAP monitoring devices.

## 5. Conclusions

According to the research guidelines of the Abdominal Compartment Society, this in vitro study shows that the TraumaGuard can be used interchangeably with the gold standard for measuring continuous IAP, even in an empty artificial bladder. Confirmation studies with the TraumaGuard in animals and humans are warranted to further validate these findings.

## Figures and Tables

**Table 1:** The Bland and Altman analysis results for the continuous 48 h (*n* = 2880) and intermittent (*n* = 300) IAP readings performed with each technology.

Study Method	Mean IAP (mm Hg)	Bias (mm Hg)	Precision (mm Hg)	LLA (mm Hg)	ULA (mm Hg)	PE (%)
48 h of continuous IAP tracings (*n* = 2880)						
CiMON	22.74	+0.71	1.75	−2.72	+4.14	15.39
Spiegelberg	22.96	+0.93	1.90	−2.79	+4.65	16.55
Serenno	22.32	+0.29	1.40	−2.45	+3.03	12.54
TraumaGuard	22.17	+0.14	1.40	−2.60	+2.88	12.62
Accuryn	21.97	−0.06	1.90	−3.80	+3.66	17.29
Intermittent IAP readings (*n* = 300)						
CiMON	19.75	+0.87	0.54	−0.19	+1.93	5.46
Spiegelberg	20.02	+1.15	0.76	−0.34	+2.63	7.59
Serenno	19.27	+0.39	0.40	−0.39	+1.17	4.15
TraumaGuard	19.13	+0.25	0.92	−1.55	+2.05	9.61
Accuryn	19.05	+0.17	0.51	−0.83	+1.17	5.35

**Table 2:** Error grid analysis results. The associated risk levels of each equipment and each risk region are presented in this table.

	No-Risk Region	Low-Risk Region	Medium-Risk Region	High-Risk Region
CiMON	92.93%	5.20%	1.04%	0.83%
Spiegelberg	92.04%	5.65%	1.45%	0.86%
Serenno	93.38%	5.03%	1.18%	0.41%
TraumaGuard	93.32%	5.06%	1.21%	0.41%
Accuryn	93.13%	5.21%	1.14%	0.52%

**Table 3:** Numerical values of the TAT and AUC for different IAP intervals. AUC: area under the curve, IAP: intra-abdominal pressure, TAT: time above a threshold, TG: TraumaGuard. *p*-values calculated with paired Student’s *t*-test. NS: Not significant.

	TAT (min)			TAT (%)		
	IAPgold	IAPTG	*p*-Value	IAPgold	IAPTG	*p*-Value
**<12**	118.5 ± 63.4	115.5 ± 63.5	NS	52.2 ± 3.6	52.3 ± 3.8	NS
**>12**	600 ± 63.3	603 ± 63.4	NS	47.8 ± 3.7	47.7 ± 3.8	NS
**12–15**	84 ± 15.2	41 ± 21.7	0.09	11.1 ± 0.5	11.0 ± 0.5	0.09
**15–20**	158.0 ± 58.1	141.8 ± 14.2	NS	15.2 ± 1.0	15.4 ± 1.0	NS
**20–25**	108.8 ± 22.3	147.8 ± 33.5	NS	10.3 ± 1.0	10.5 ± 0.9	NS
**>25**	250 ± 39.6	273.3 ± 62.6	NS	11.2 ± 2.8	10.9 ± 2.8	NS
	**AUC (min × mm Hg)**			**AUC (%)**		
**<12**	8244.4 ± 139.9	8334.6 ± 104.9	0.05	52.2 ± 3.6	63.5 ± 3.8	NS
**>12**	7599.2 ± 1026.9	7649.7 ± 1078	NS	47.8 ± 3.7	63.4 ± 3.8	NS
**12–15**	1754.5 ± 139.3	1754.4 ± 140.1	NS	11.1 ± 0.5	21.7 ± 0.5	NS
**15–20**	2411.0 ± 238.9	2457.5 ± 257	NS	15.2 ± 1.0	14.2 ± 1.0	NS
**20–25**	1635.6 ± 243.5	1676.6 ± 250.8	0.05	10.3 ± 1.0	33.5 ± 0.9	NS
**>25**	1798.0 ± 540.1	1761.3 ± 553.7	NS	11.2 ± 2.8	62.6 ± 2.8	0.02

**Table 4:** Further information extracted from dynamic IAP tracings. IAP_ei_, IAP_ee_, APV, mean IAP, ΔIAP, inspiration-to-expiration ratio, respiration, and heart rate were calculated for each technology. The results are presented based on the calculations of five raw IAP tracings.

	Reference	CiMON	Spiegelberg	Serenno	TraumaGuard	Accuryn
IAP_ei_ (mm Hg)	25	25.88 ± 0.30	26.07 ± 0.08	26.29 ± 0.07	25.15 ± 0.22	25.72 ± 0.13
IAP_ee_ (mm Hg)	20	20.7 ± 0.20	21.59 ± 0.11	21.23 ± 0.47	20.27 ± 0.18	21.63 ± 0.07
Mean IAP (mm Hg)	22.5	23.29 ± 0.15	23.83 ± 0.09	23.76 ± 0.23	22.71 ± 0.13	23.78 ± 0.10
ΔIAP (mm Hg)	5	5.18 ± 0.41	4.48 ± 0.10	5.06 ± 0.48	4.88 ± 0.31	4.07 ± 0.27
APV (%)	22.22	22.24 ± 1.75	18.79 ± 0.29	21.27 ± 1.48	21.48 ± 1.01	17.20 ± 0.32
HR (bpm)	120	127.11 ± 4.59	84.04 ± 7.32	98.21 ± 6.48	125.16 ± 6.11	87.12 ± 8.41
RR (rpm)	16.21	16.07 ± 0.71	16.46 ± 0.84	17.05 ± 0.52	15.80 ± 0.85	16.14 ± 0.64
T_insp_ (sec)	1.8	1.79 ± 0.12	1.79 ± 0.09	1.70 ± 0.12	1.86 ± 0.21	1.83 ± 0.02
T_exp_ (sec)	1.9	1.96 ± 0.24	1.86 ± 0.13	1.80 ± 0.07	1.95 ± 0.04	1.88 ± 0.21
I/E (ratio)	0.95	0.93 ± 0.17	0.96 ± 0.02	0.96 ± 0.09	0.95 ± 0.11	0.97 ± 0.13

IAP_ei_: end-inspiration IAP, IAP_ee_: end-expiration IAP, APV: abdominal pressure variation, HR: heart rate, RR: respiration rate, T_insp_: inspiration time, T_exp_: expiration time, I/E: inspiration-to-expiration time ratio.

**Table 5:** An overview of the investigated IAP measurement systems. Taking the bias and the standard deviation of bias of each technology into account, we compared the accuracy and precision, respectively. According to the dynamic tracing analysis of each device, the ability to track respirations and heartbeats was evaluated as well. The zeroing procedure is automatic except for in the TraumaGuard. Additionally, the risk level, temperature, and bladder fill volume dependency of each system were compared with the others based on the results of the error grid analysis and the temperature and bladder fill volume experiments.

Parameter	Intra-Gastric		Intra-Bladder		
	CiMON	Spiegelberg	Serenno	Accuryn	TraumaGuard
Zeroing Procedure (autocalibration)	5	5	5	5	4
Accuracy	4	4	5	5	5
Precision	4	4	5	4	4
Respiration monitoring	5	5	5	4	5
Heartbeat monitoring	5	4	4	4	4
Associated risk level	5	5	5	5	5
Temperature dependency	5	3	5	5	5
Data extraction capability	5	5	4	3	5
Bladder fill volume dependency	NA	NA	3	3	5
Average in vitro score (scale from 1 to 5)	4.75 ± 0.43	4.38 ± 0.69	4.50 ± 0.68	4.22 ± 0.78	4.67 ± 0.43

## Data Availability

The derived data supporting the findings of this study, in addition to the processing algorithms, are available from the corresponding author upon request.

## Supplementary Materials

The following supporting information can be downloaded at: https://www.mdpi.com/article/10.3390/jcm12196260/s1, Table S1: Mean and standard deviation of each system at pre-set IAP values of 0, 5, 10, 15, 20, 25, 30, 35, and 40 mm Hg; Figure S1: Artificial bladder of the abdominal phantom; Figure S2: Relative error in IAP measurements obtained with each equipment at room and body temperature; Figure S3: Correlation matrix; Figure S4: Intra-class correlation analysis results of the continuous 48 h IAP measurement; Figure S5: Bland and Altman plots according to 300 intermittent IAP readings; Figure S6: Concordance (4-quadrant) plots for (**a**) CiMON, (**b**) Spiegelberg, (**c**) Serenno, (**d**) TraumaGuard, and (**e**) Accuryn during 48 h continuous IAP monitoring (*n* = 2880); Figure S7: Concordance (4-quadrant) plots for 300 intermittent IAP measurements. Figure S8: Four sets of 12 h continuous IAP measurement (CiMON); Figure S9: Four sets of 12 h continuous IAP measurement (Spiegelberg); Figure S10: Four sets of 12 h continuous IAP measurement (Serenno); Figure S11: Four sets of 12 h continuous IAP measurement (Accuryn); Figure S12: Dynamic IAP tracings captured by the studied systems.
