# Supplementary material for: In Vitro Validation of a Novel Continuous Intra-Abdominal Pressure Measurement System (TraumaGuard)
Source: J Clin Med. 2023 Sep 28;12(19):6260. doi: 10.3390/jcm12196260 (PMC10573363; doi:10.3390/jcm12196260)
Supplement: Supplementary file 1 [file jcm-12-06260-s001.zip › Table S1.pdf]

IAP_{H2O} [mmHg] (reference IAP)	0	5	10	15	20	25	30	35	40
**IAP_{CiM} [mmHg]**	1.27 ± 0.15	6.32 ± 0.23	11.17 ± 0.25	16.11 ± 0.26	20.65 ± 0.27	25.38 ± 0.18	30.45 ± 0.22	34.8 ± 0.42	39.36 ± 0.31
**IAP_{Spie} [mmHg]**	2.45 ± 0.79	6.23 ± 0.29	10.75 ± 0.62	16.06 ± 0.35	20.61 ± 0.42	25.89 ± 0.29	30.85 ± 0.30	35.65 ± 0.24	40.6 ± 0.25
**IAP_{Ser}[mmHg]**	0.42 ± 0.12	5.14 ± 0.32	9.86 ± 0.61	14.85 ± 0.58	19.80 ± 0.76	24.68 ± 0.59	29.93 ± 0.64	34.76 ± 0.50	39.97 ± 0.62
**IAP_{TG} [mmHg]**	0.81 ± 0.48	5.73 ± 0.85	10.67 ± 0.75	15.72 ± 0.61	20.37 ± 0.46	24.96 ± 0.66	30.03 ± 0.72	34.57 ± 0.57	39.32 ± 0.35
**IAP_{Acc} [mmHg]**	0.04 ± 0.03	4.98 ± 0.13	10.12 ± 0.35	15.20 ± 0.29	20.10 ± 0.37	25.12 ± 0.26	30.05 ± 0.25	35.22 ± 0.24	40.20 ± 0.49
