# Supplementary figures and images for: In Vitro Validation of a Novel Continuous Intra-Abdominal Pressure Measurement System (TraumaGuard)
Source: J Clin Med. 2023 Sep 28;12(19):6260. doi: 10.3390/jcm12196260 (PMC10573363; doi:10.3390/jcm12196260)

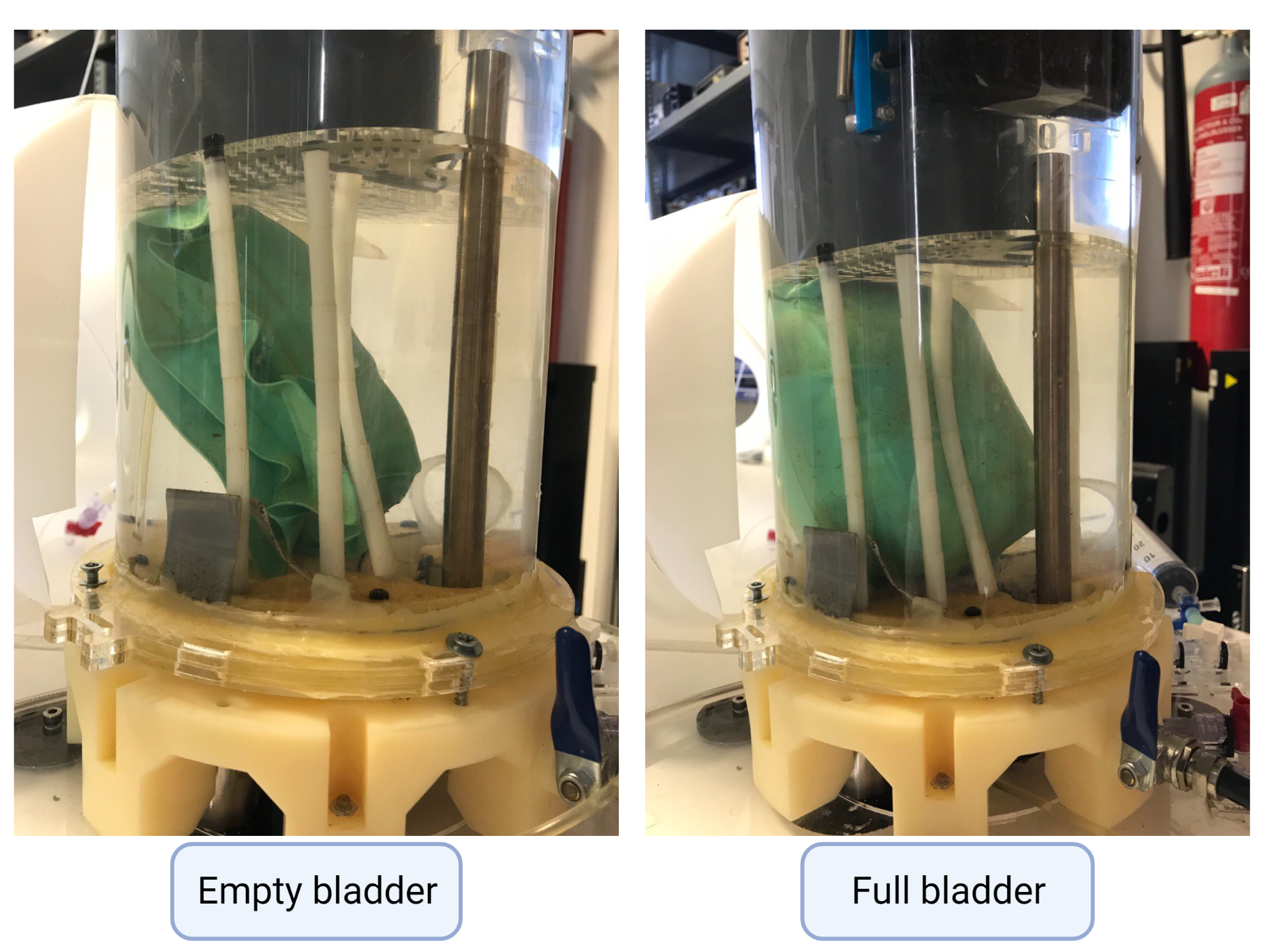

Supplement: Supplementary file 1 [file jcm-12-06260-s001.zip › Figure S1.tif]

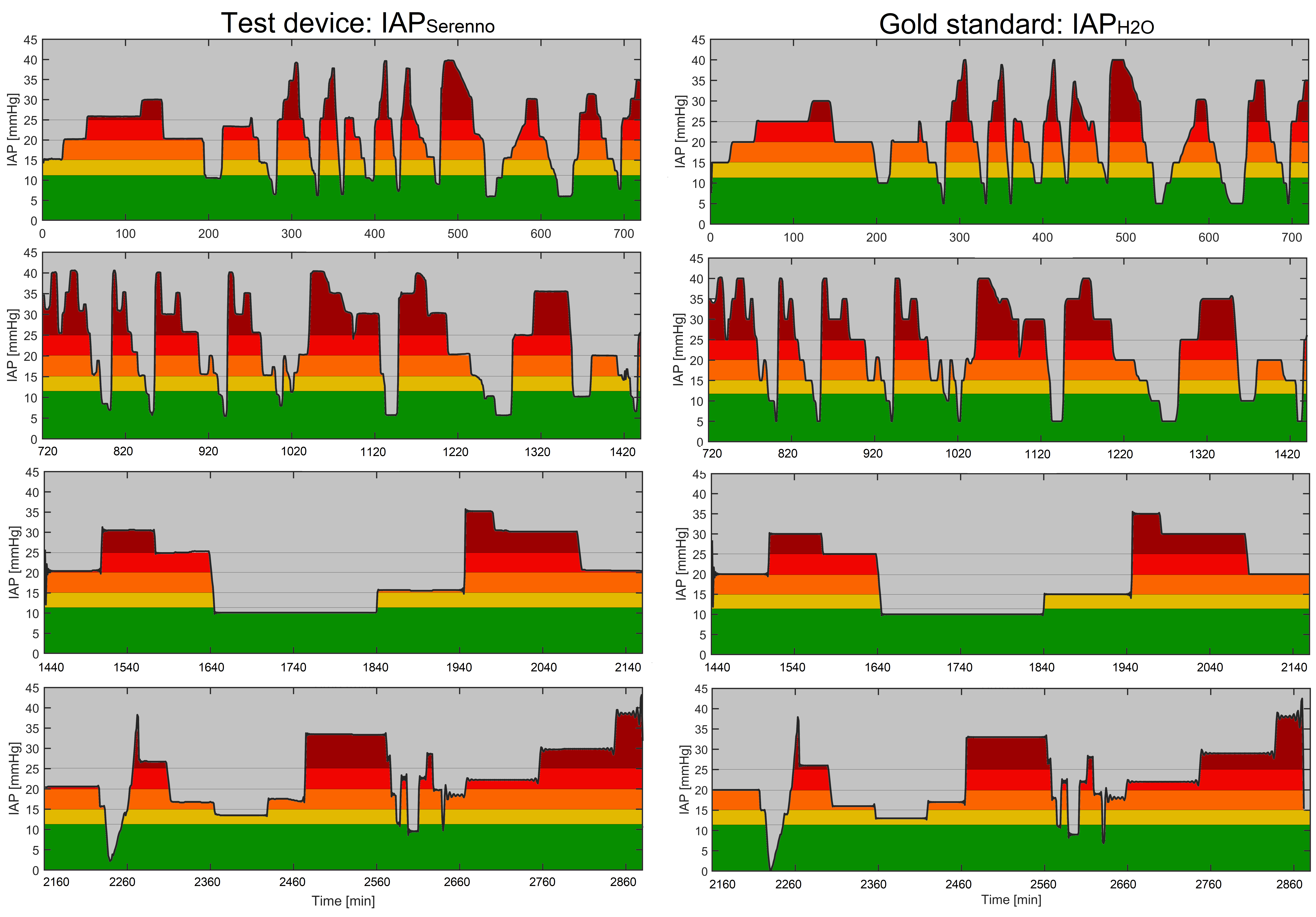

Supplement: Supplementary file 1 [file jcm-12-06260-s001.zip › Figure S10.tif]

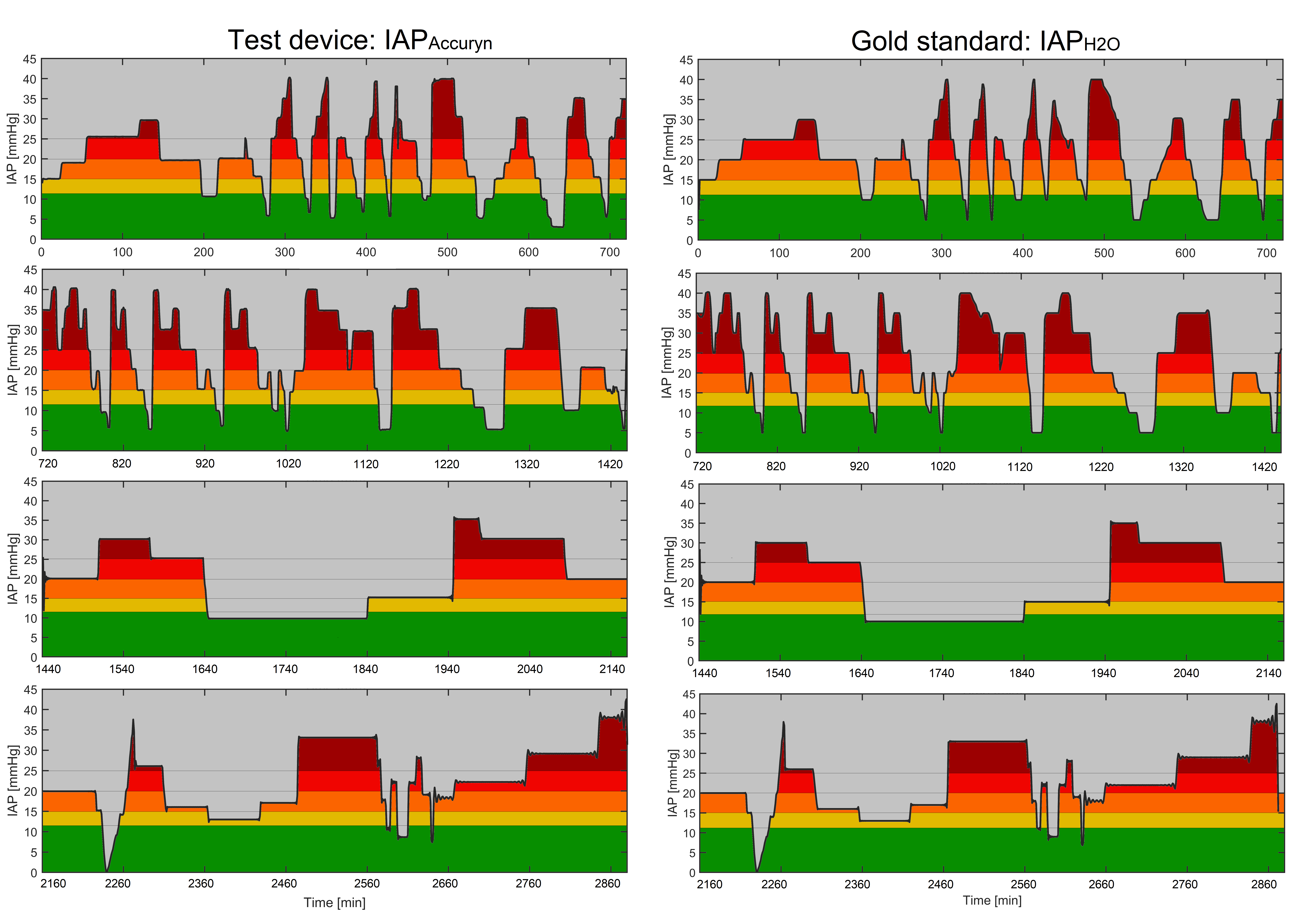

Supplement: Supplementary file 1 [file jcm-12-06260-s001.zip › Figure S11.tif]

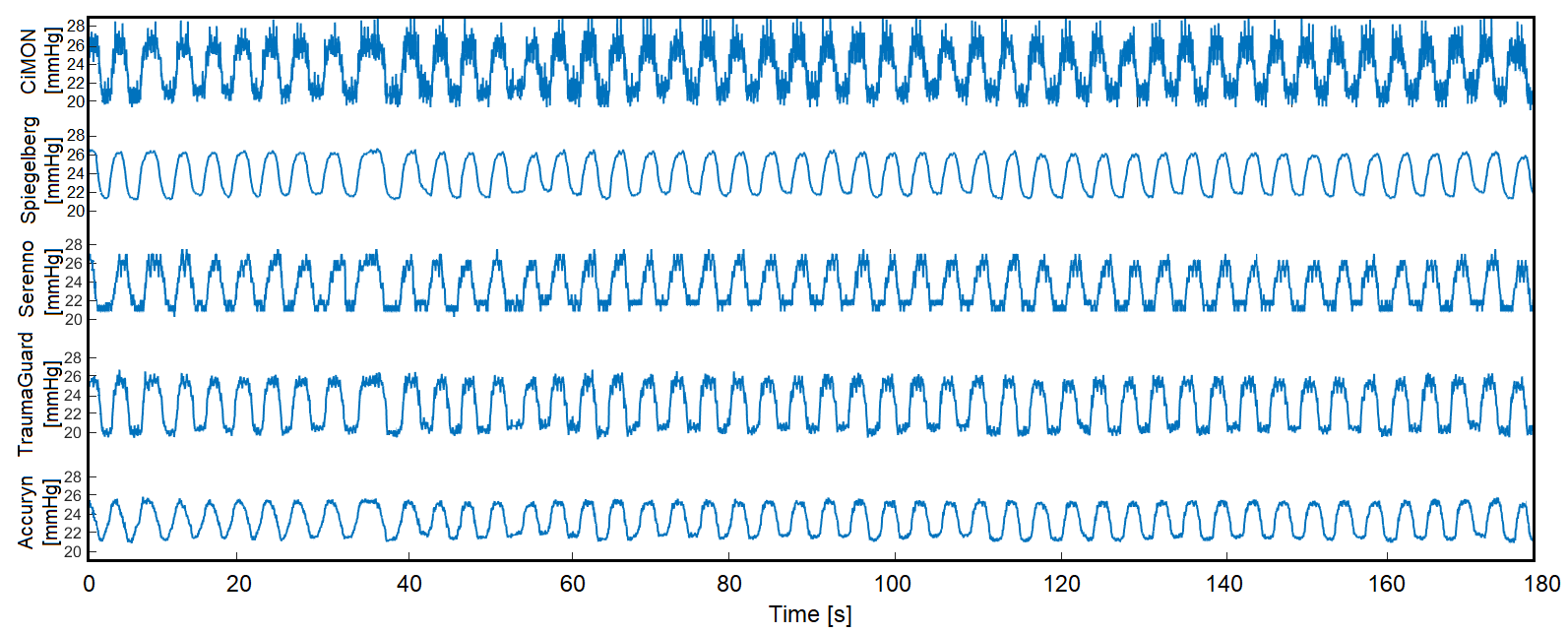

Supplement: Supplementary file 1 [file jcm-12-06260-s001.zip › Figure S12.tif]

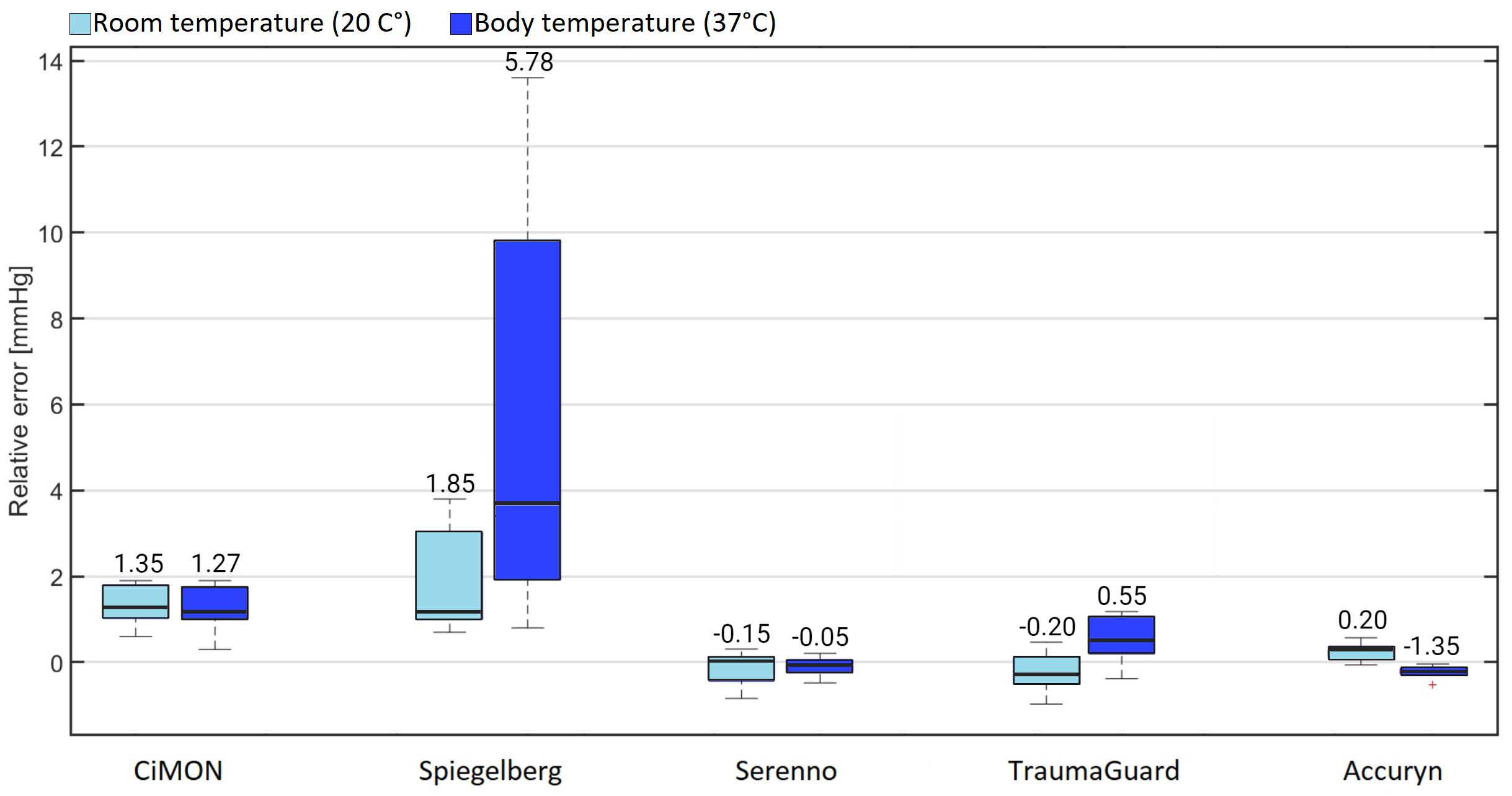

Supplement: Supplementary file 1 [file jcm-12-06260-s001.zip › Figure S2.tif]

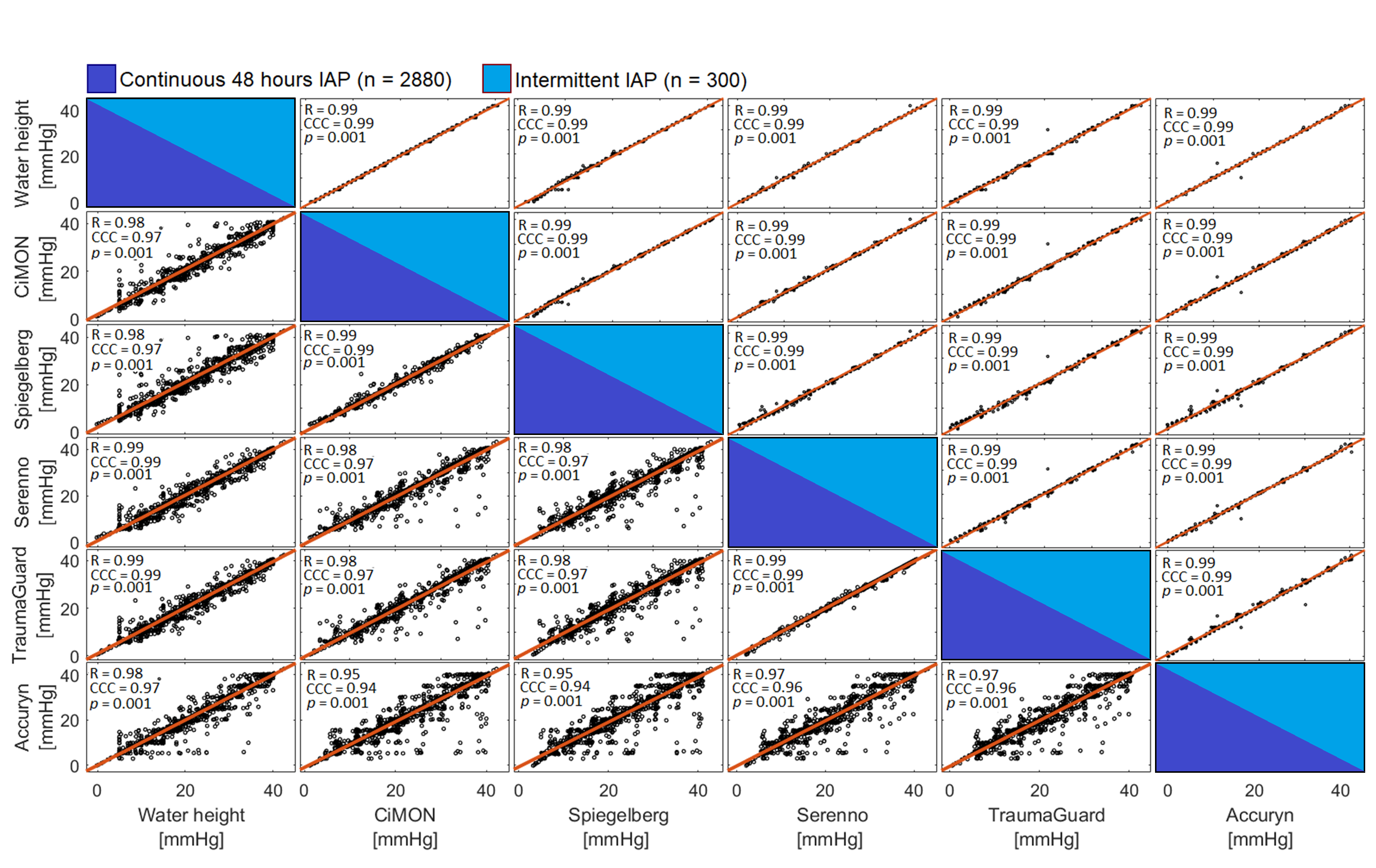

Supplement: Supplementary file 1 [file jcm-12-06260-s001.zip › Figure S3.tif]

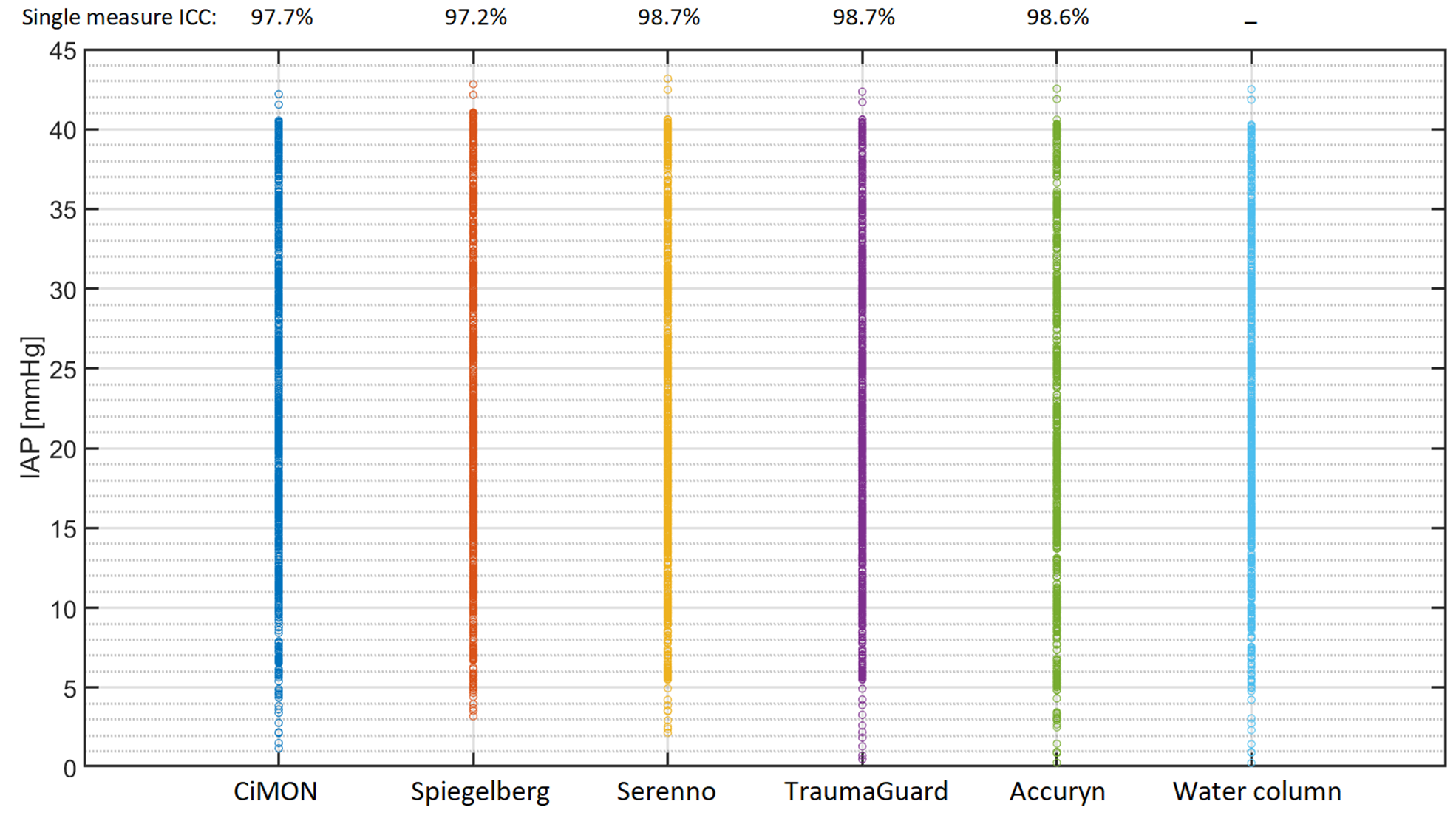

Supplement: Supplementary file 1 [file jcm-12-06260-s001.zip › Figure S4.tif]

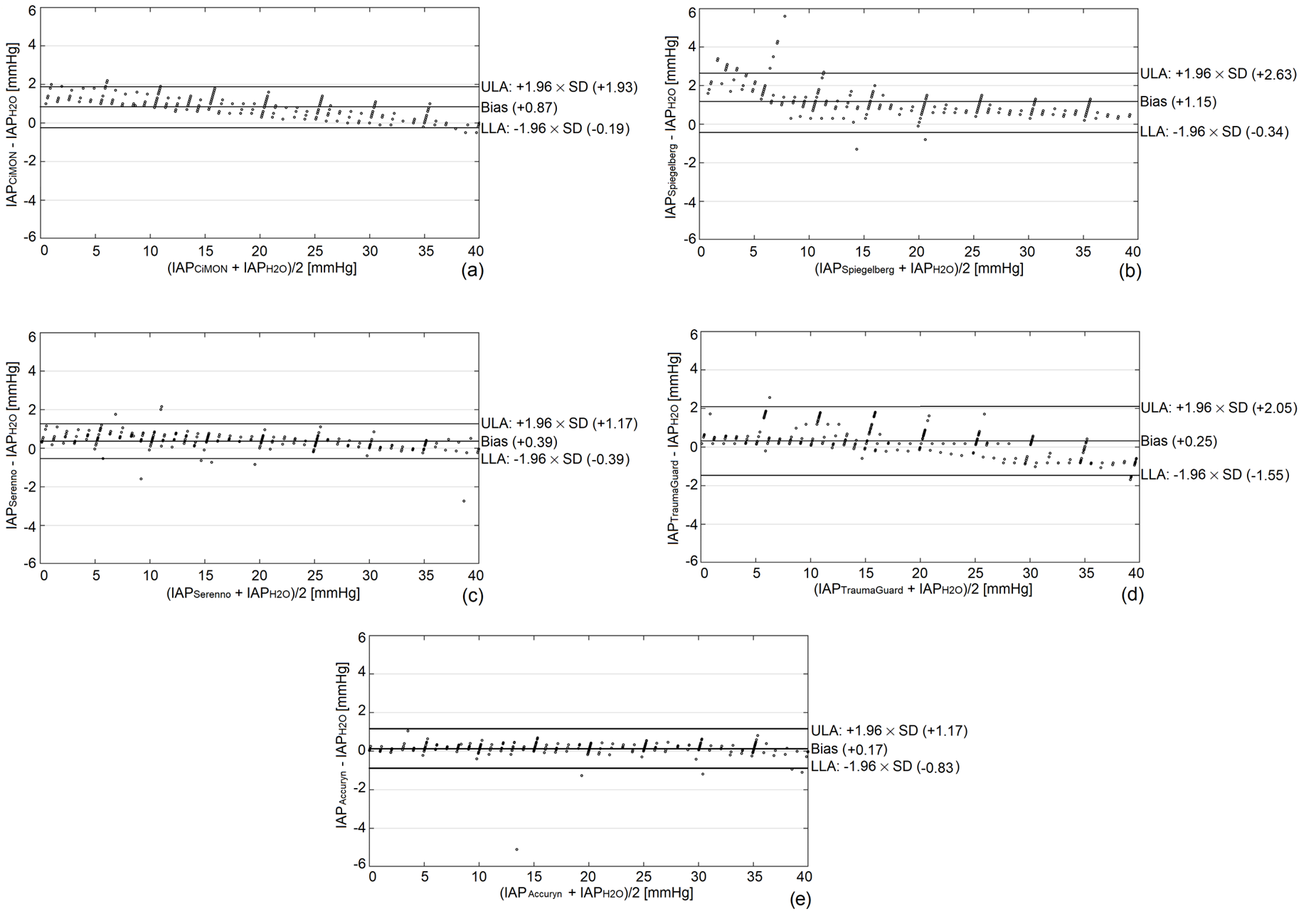

Supplement: Supplementary file 1 [file jcm-12-06260-s001.zip › Figure S5.tif]

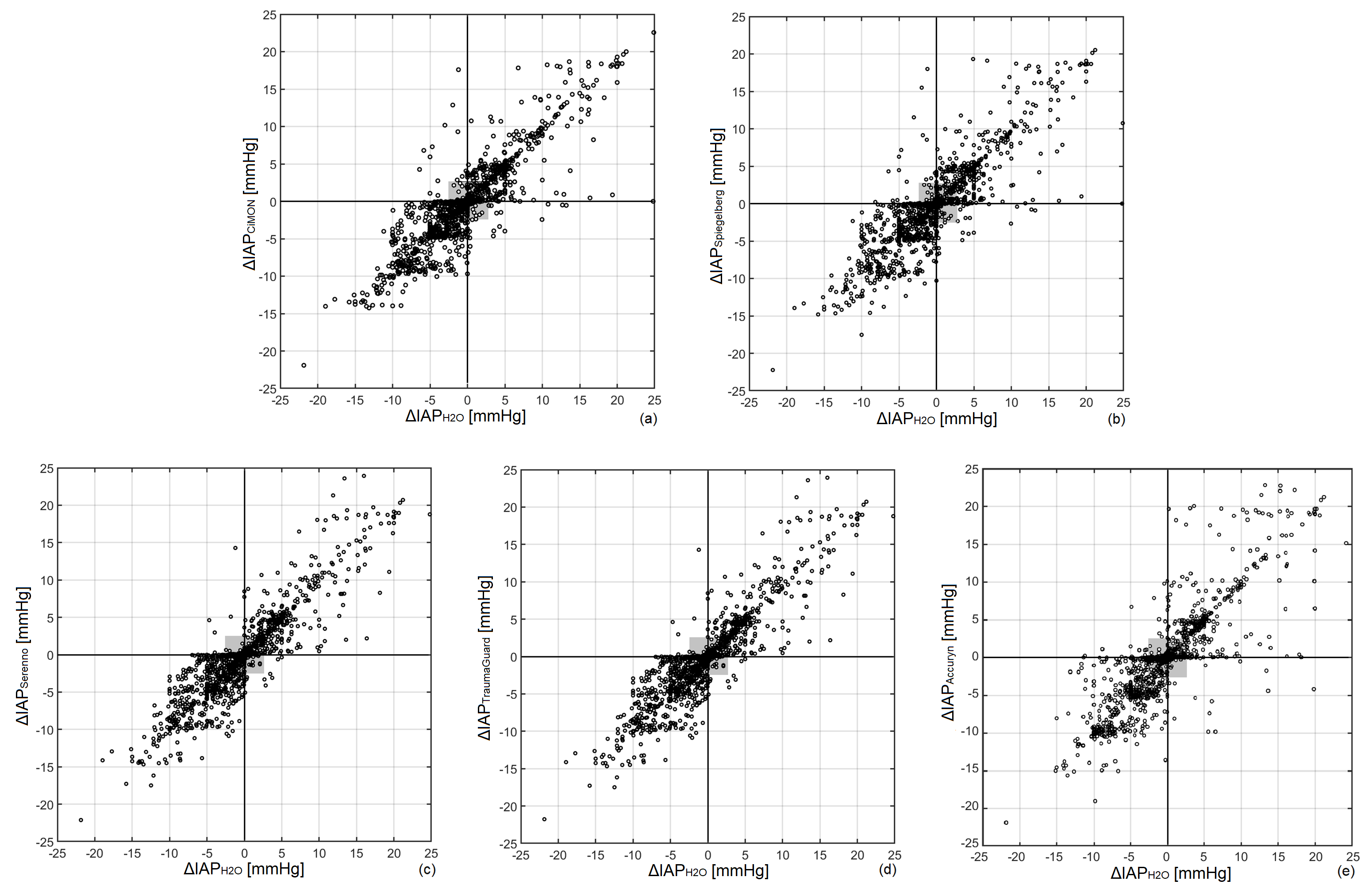

Supplement: Supplementary file 1 [file jcm-12-06260-s001.zip › Figure S6.tif]

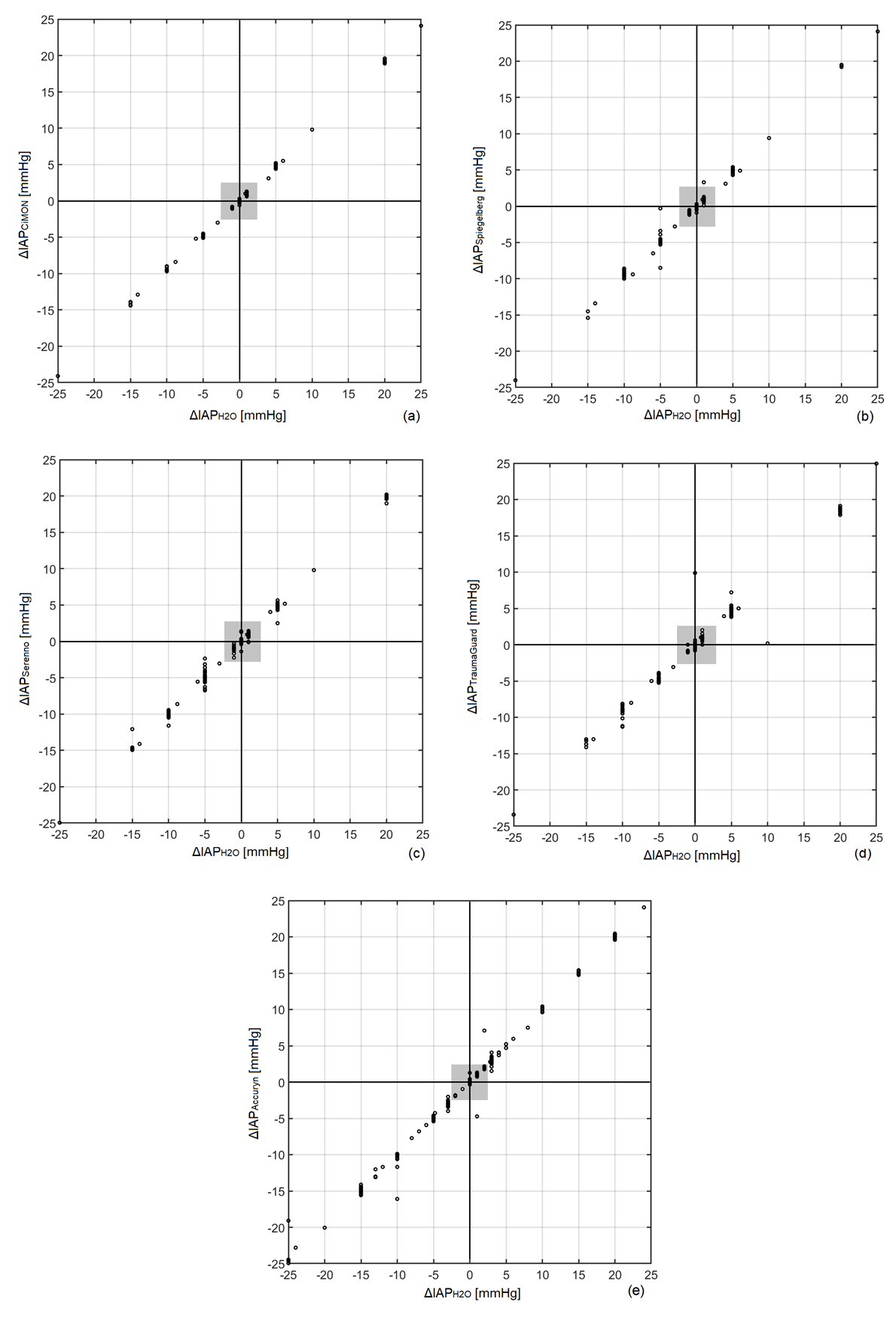

Supplement: Supplementary file 1 [file jcm-12-06260-s001.zip › Figure S7.tif]

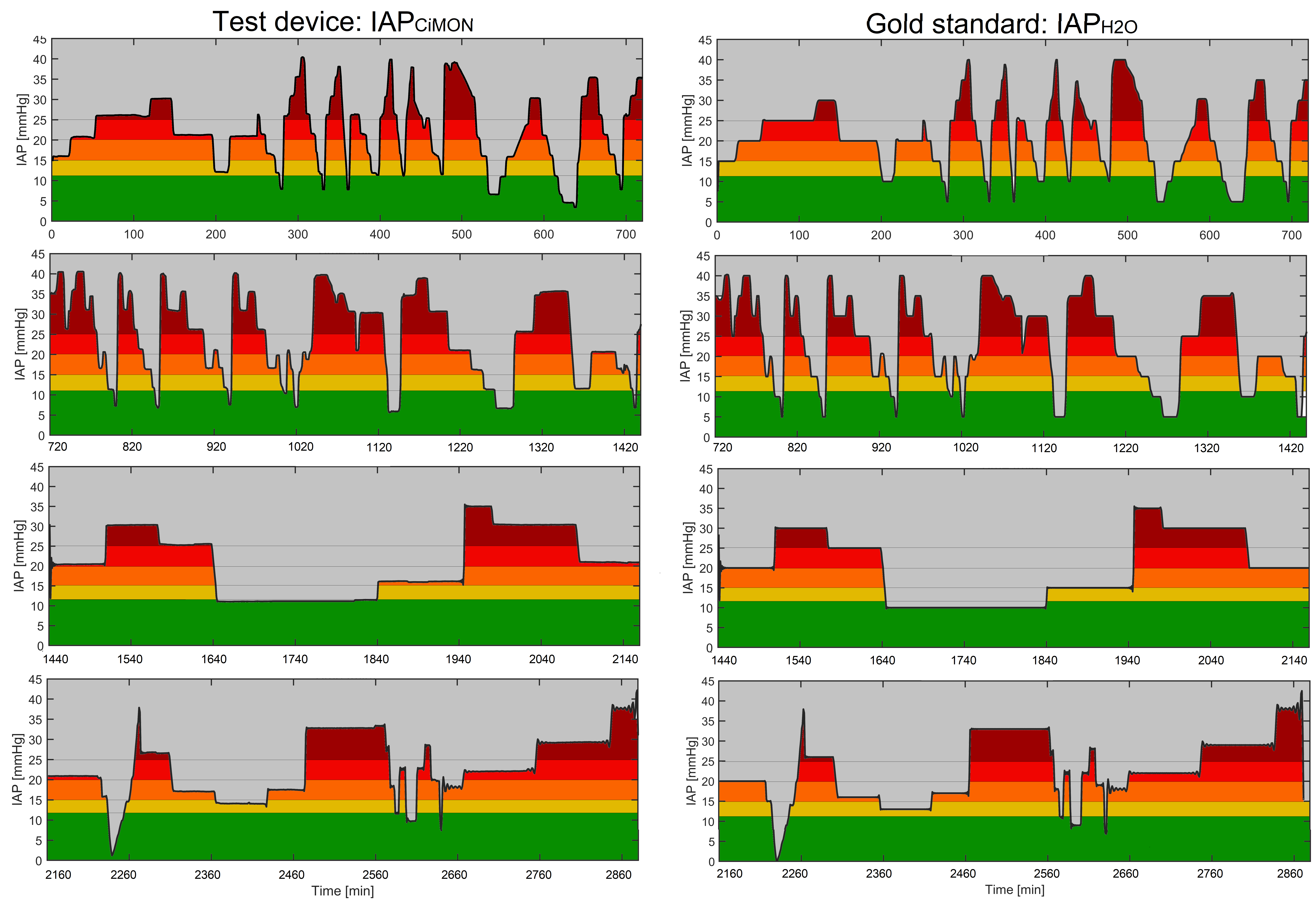

Supplement: Supplementary file 1 [file jcm-12-06260-s001.zip › Figure S8.tif]

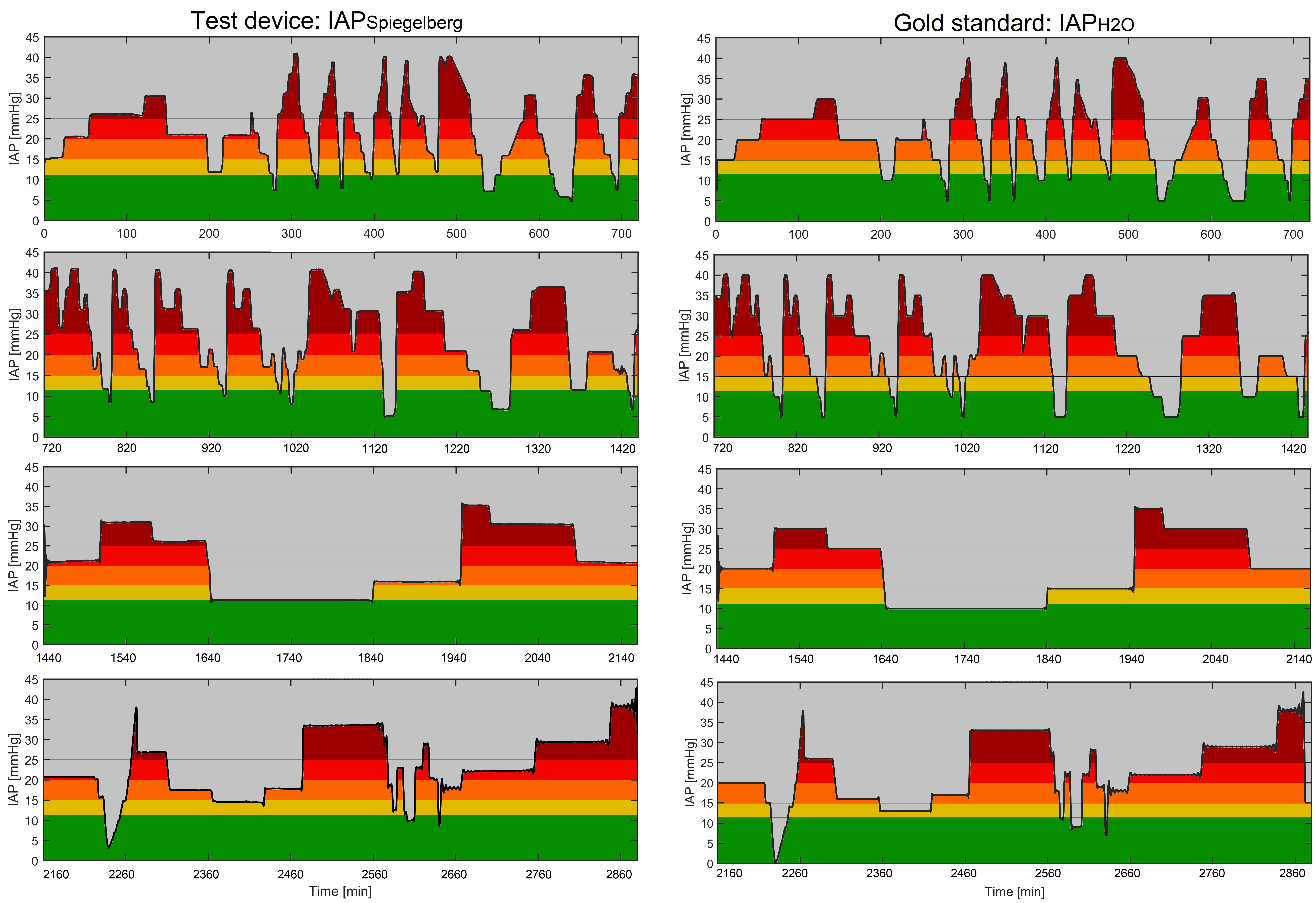

Supplement: Supplementary file 1 [file jcm-12-06260-s001.zip › Figure S9.tif]
